# *Salmonella* Gallinarum delivering M2eCD40L in protein and DNA formats acts as a bivalent vaccine against fowl typhoid and H9N2 infection in chickens

**DOI:** 10.1186/s13567-018-0593-z

**Published:** 2018-10-01

**Authors:** Irshad Ahmed Hajam, Jehyoung Kim, John Hwa Lee

**Affiliations:** College of Veterinary Medicine, Chonbuk National University, Iksan, 54596 Republic of Korea

## Abstract

Fowl typhoid (FT), a septicemic disease caused by *Salmonella* Gallinarum (SG), and H9N2 influenza infection are two economically important diseases that affect poultry industry worldwide. Herein, we exploited a live attenuated SG mutant (JOL967) to deliver highly conserved extracellular domains of H9N2 M2 (M2e) to induce protective immunity against both H9N2 infection and FT. To increase the immunogenicity of M2e, we physically linked it with CD40L and cloned the fusion gene into either prokaryotic constitutive expression vector pJHL65 or mammalian expression vector pcDNA3.1+. Then pJHL65-M2eCD40L or pcDNA-M2eCD40L recombinant plasmid was electroporated into JOL967 strain and the resultant clones were designated as JOL2074 and JOL2076, respectively. We demonstrated that the chickens vaccinated once orally with a co-mix of JOL2074 and JOL2076 strains elicited significantly (*p* < 0.05) higher M2e-specific humoral and cell-mediated immunity compared to JOL2074 alone vaccinated group. However, SG-specific immune responses were comparable in both the vaccination groups. On challenge with the virulent H9N2 virus (10^5 ^TCID_50_) at 28^th^ day post-vaccination, chickens that received a co-mix of JOL2074 plus JOL2076 strains exhibited significantly (*p* < 0.05) lower lung inflammation and viral load in both lungs and cloacal samples than JOL2074 alone vaccinated group. Against challenge with the lethal wild-type SG, both the vaccination groups exhibited only 12.5% mortality compared to 75% mortality observed in the control group. In conclusion, we show that SG delivering M2eCD40L can act as a bivalent vaccine against FT and H9N2 infection and further studies are warranted to develop this SG-M2eCD40L vaccine as a broadly protective vaccine against avian influenza virus subtypes.

**Electronic supplementary material:**

The online version of this article (10.1186/s13567-018-0593-z) contains supplementary material, which is available to authorized users.

## Introduction

Avian influenza (AI) and fowl typhoid are two highly contagious infectious diseases that cause huge economic losses in poultry industry worldwide [1, 2]. Avian influenza viruses (AIV), the causative agents of AI, have worldwide distribution in domestic and wild poultry and are broadly designated as high-pathogenicity avian influenza (HPAI) or low-pathogenicity avian influenza (LPAI) based on the pathogenicity and the virulence in chickens [3, 4]. H9N2, a LPAI virus, is the most widespread influenza subtype circulating endemically in poultry across Asia, the Middle East and Northern Africa [5, 6]. Despite being low pathogenic in nature, H9N2 outbreaks result in huge economic losses in poultry industry, largely due to reduced egg production, reduced feed conversion efficiencies and highly lethal secondary bacterial or viral co-infections [7, 8]. Being the primarily poultry pathogen, H9N2 internal gene cassette (polymerase genes, nucleoprotein, matrix and non-structural genes) has been reported to diversify the host range and conferring zoonotic transmission potential to non-H9N2 influenza strains, with recent examples including H7N9 and H10N8 outbreaks in china that resulted in human fatalities [9–11]. Thus, H9N2 strains can lead to the emergence of novel reassortants with ability to cause potential pandemics. Additionally, H9N2 sporadic human infections have been reported in the past [12, 13], suggesting that this influenza subtype has zoonotic potential. Therefore, the development of an effective vaccine to prevent and control H9N2 infection in poultry has an immense public health importance. In South Korea, H9N2 subtype has been endemic since 2000 and the infection is mainly controlled through the use of an oil adjuvanted inactivated H9N2 vaccine, which has dramatically decreased the incidence of H9N2 infection in chicken farms [14]. However, these conventional vaccines require a large supply of specific-pathogen-free (SPF) embryonated eggs and vaccine production requires a long timeline that could be threatened during pandemic situations. Furthermore, these conventional vaccines mainly provide homotypic protection with little cross protective immunity as vaccine strains should closely match to the circulating field strains. The introduction and development of an effective vaccine against each LPAI subtype is not an economically viable option for the poultry industry. Thus, novel approaches should be devised that are not only egg independent and cost-effective, but easy to amplify and broadly protective against LPAI viruses, particularly H9N2.

Similar to hemagglutinin and neuraminidase, the ectodomain of matrix protein 2 (M2e) is an integral transmembrane protein of Influenza A viruses and is considered as a promising candidate antigen to elicit cross-protective immunity [15]. It is well documented that M2e is highly conserved among influenza A viruses and animal experiments have shown that M2e-specific antibodies can provide broad spectrum of protection against different types of influenza A subtypes [15, 16]. While natural infections and currently available conventional influenza vaccines induce very weak M2e-specific immunity, presenting M2e on a suitable carrier molecule greatly enhances M2e-specific immunogenicity and cross-protective potential (reviewed in [15]). CD40 ligand (CD40L), a member of the tumor necrosis factor superfamily, is primarily expressed on the surface of activated T cells and binds to CD40 on antigen presenting cells (APCs), which leads to many effects including maturation of APCs and elicitation of efficient T cell responses [17]. The CD40-CD40L interactions are essential for efficient induction of primary and secondary CD8+ cytotoxic T lymphocyte responses [17]. A previous study by Ninomiya et al. [18] demonstrates that coadministration of a synthetic NP366-374 peptide encapsulated in liposome together with an anti-CD40 antibody elicited protective immunity against influenza A virus. In accordance with this notion, we physically linked M2e to chicken CD40L and delivered this fusion protein via *Salmonella* Gallinarum (SG).

Fowl typhoid (FT), a septicemic disease of poultry, is caused by an intracellular Gram-negative bacterium SG, which causes acute mortality in domestic birds, primarily chickens. The disease has worldwide distribution and is endemic in many parts of the world. We previously have developed an attenuated SG vaccine strain (JOL916) that effectively controls FT infection in chickens [19]. Exploiting live attenuated bacterial system to deliver influenza antigens is a highly economical vaccination strategy that allows for a quick response to novel influenza viruses, as it circumvents the need for a constant supply of SPF embryonated eggs. Previously, a single M2e6-13 B cell epitope physically linked to the human CD40L has been stably integrated in the *Salmonella* Enteritidis genome and tested in chickens as a vaccine candidate against H7N2 virus [20]. In the present study, we physically linked M2e (consisting of two B cell epitopes and a T cell epitope) to chicken CD40L and delivered this fusion protein via SG. We hypothesized that delivering M2eCD40L in both protein and DNA formats via SG could act as a bivalent vaccine against FT and LPAI viruses in chickens. Herein, we report the construction of an attenuated auxotrophic mutant of SG delivering M2eCD40L in both protein and DNA formats and induces efficient immune protection against H9N2 and wild-type SG virulent challenges in chickens.

## Materials and methods

### Bacterial strains, plasmids, cell line and virus

The bacterial strains and plasmids used in this study are listed in Table 1. The tissue culture infective dose (TCID_50_) of H9N2 influenza A virus, cultivated in the allantoic cavities of SPF embryonated eggs, was calculated in Madin Darby Canine Kidney (MDCK) cells as previously described [21].

**Table 1:** **Bacterial strains and plasmids used in this study**

Strains/plasmids	Description	References
Strains		
Χ232	*E. coli* Δ*asd* strain, used for cloning of genes into *asd*^+^ plasmid	[28]
JOL394	*Salmonella* Gallinarum wild-type challenge strain	[28]
JOL967	*∆lon, ∆cpxR and ∆asd* mutant *of S.* Gallinarum	[28]
JOL916	*∆lon, ∆cpxR mutant of S. Gallinarum*	[28]
JOL2068	JOL967 with pJHL65 empty plasmid	[44]
JOL2074	JOL967 with pJHL65-M2eCD40L plasmid	This study
JOL2076	JOL967 with pJHL65 plus pcDNA-M2eCD40L	This study
Plasmids		
pJHL65	An *asd*^+^ vector, pBR ori, β-lactamase signal sequence-based periplasmic secretion plasmid, 6xHis, high copy number	[21]
pcDNA3.1+	Mammalian expression vector, CMV promoter, high copy number	(Invitrogen, USA)
pET-32a	IPTG-inducible expression vector; Kanamycin resistant	(Novagen, USA)
pET32a-M2eCD40L	M2e-CD40L gene in pET32a vector	This study

### Construction of an attenuated SG mutant delivering M2eCD40L in protein and DNA formats

The conserved H9N2 M2e gene sequence (MSLLTEVETPTRNGWECKCSDSSD) physically linked to the avain CD40 ligand (CD40L) peptide sequence (WMTTSYAPTSS, accession no. NP_990064.1) was chemically synthesized (Bionee, Korea) and built into the pJHL65 plasmid, an *asd*+ constitutive expression vector, and propagated in an *asd* mutated *Escherichia coli* strain as described previously [22]. The M2eCD40L sequence was cloned in frame downstream to the beta-lactamase signal sequence (*bla SS*) so that the intended protein would get secreted out of the bacteria [23]. The M2eCD40L fusion gene was also cloned into a mammalian expression vector, pcDNA3.1+ (Invitrogen, USA), and propagated in *E. coli* DH5-alpha strain (Invitrogen, USA). The pJHL-M2eCD40L and pcDNA-M2eCD40L recombinant plasmids were subsequently electroporated into JOL967 and JOL2068 auxotrophic mutant strains of SG, respectively, and the resultant clones were designated as JOL2074 and JOL2076, respectively. The JOL967 strain was attenuated by deletion of *lon*, *cpxR*, and *asd* genes from the wild-type SG, JOL394 isolate, by an allelic exchange method described and reported elsewhere [19], while JOL2068 is JOL967 strain carrying an empty *asd*+ plasmid. The expression of M2eCD40L displayed on the surface of SG was confirmed by an indirect immunofluorescence method as described previously [24], following incubation of mid-logarithmic grown bacteria (OD_600 nm_ = 0.6) with primary M2e-specific polyclonal antibody (catalog#, MBS9405612) and then labelling with PE-conjugated species-specific secondary antibody (catalog#, STAR12A). The labelled bacteria were examined by light microscopy (Axio Imager 2, Zeiss, Germany) and digital imaging software (Axio Vision, Zeiss, Germany). We further validated M2eCD40L expression by flow cytometry as previously described [25], following incubation of bacteria first with primary M2e-specific polyclonal antibody and then labelling with Alexa Fluor 488-conjugated secondary antibody (catalog#, A21206). The bacteria displaying surface M2eCD40L exhibiting fluorescence were gated and the results were expressed in percentage of bacteria showing expression. We also confirmed the expression of M2eCD40L in *E. coli* BL21 (DE3) pLysS host strain (Novagen, San Diego, USA) by cloning M2eCD40L gene sequence in pET32a (+) expression vector as previously described [22]. The protein expressed by BL21 strain was purified and dialysed against PBS, and stored at −80 °C until further use.

To investigate whether M2eCD40L have any effect on the growth of SG, we evaluated the growth kinetics of JOL2068, JOL2074 and JOL2076 by growing each strain in LB broth at 37 °C and the growth pattern of each strain was monitored by measuring the optical density (OD600 nm).

### Gene delivery and expression of M2eCD40L in cultured cells mediated by JOL2076

Next we investigated the transfer of pcDNA-M2eCD40L DNA plasmid mediated by JOL2076 into mammalian cells keeping LyoVec™ (InvivoGen, San Diego, USA) mediated transfer of pcDNA-M2eCD40L plasmid into MDCK cells as positive control. In gene transfer experiment, we infected MDCK cells (1 × 10^6^ cells) with SG mutant (JOL2076) delivering M2eCD40L DNA at a multiplicity of infection (MOI) of 20 bacteria/cell and the infected culture was then incubated at 37 °C in 5% CO_2_ for 1 h. Thereafter, the cells were washed five times with phosphate-buffered saline (PBS) and then incubated with fresh medium containing 50 μg/mL of gentamicin for 30 h. LyoVec™ mediated delivery of pcDNAM2eCD40L into MDCK cells was done as per the manufacturers’ instructions. Since the size of M2eCD40L fusion protein is approximately 4 kDa which is too small for the Western blot analysis, so we validated the expression of M2eCD40L protein at mRNA level by qRT-PCR assay. A standard curve was made by serially diluting pcDNA-M2eCD40L plasmid in qRT-PCR assay and the mRNA copy number of M2eCD40L fusion protein expression was calculated [21].

### Immunization and challenge studies

All animal experimentation work was approved by the Chonbuk National University Animal Ethics Committee (CBNU2015-00085) and the chicken experiment was carried out according to the guidelines of the Korean Council on Animal Care. One-day-old female brown nick layer chickens (Corporation of Join hatchery, Republic of Korea) were maintained under standard conditions and provided antibiotic-free food and water ad libitum. Four weeks later, the chickens were randomly divided into four groups (*n *= 10) and immunized once orally with JOL2068 (SG-vector), JOL2074 (SG-M2eCD40L), JOL2074 plus JOL2076 (SG-pcDNA-M2e) or 100 µL of sterile PBS. The 10^9^ colony forming units (CFU) of each bacterial strain were used for inoculation in this study. Blood (2 mL) was drawn from jugular vein of 5 randomly vaccinated and control chickens on day 28 post-vaccination for isolation of serum and peripheral blood mononuclear cells (PBMCs) as described previously [21]. To assess mucosal secretory (s) IgA responses, three chickens in each group were sacrificed on day 28 post-vaccination and lung and intestinal wash samples were collected.

Four weeks post-vaccination, all the immunized and the control chickens were intranasally challenged with 10^5^ TCID_50_ of H9N2 influenza A virus. The cloacal swab samples were collected at 1, 3 and 6 days post-challenge as described previously [26]. Subsequently, total RNA was isolated from the cloacal swab samples to determine the H9N2 viral load by qRT-PCR assay as described previously [21]. For histopathological and immunohistochemistry analysis, three and four chickens in each group were sacrificed on day 3 and 7 post-challenge, respectively. Lung tissues were aseptically collected and fixed with 10% formalin, embedded in paraffin and cut into 5 μm thin sections which were subsequently processed for histopathological and immunohistochemistry analysis. For histopathological analysis, sections were stained with hematoxylin and eosin (H&E) and examined by light microscopy (Axio Imager 2, Zeiss, Germany) and digital imaging software (Axio Vision, Zeiss, Germany). Immunohistochemical staining of the sectioned tissue samples using H9N2 HA-specific polyclonal antibody (catalog#, MBS270809) was performed as described previously [27]. We also separately maintained four groups (*n* = 8) immunized once orally with attenuated SG mutant (JOL916), JOL2074, JOL2074 plus JOL2076 or sterile PBS, and 4 weeks later, challenged orally with 100 µL of a suspension containing 1.6 × 10^6^ CFU of a wild-type SG (JOL394 strain) as described previously [28]. The protective efficacy of the SG-M2e based vaccine against JOL394 was evaluated in the context of the mortality of the challenged birds, which was monitored daily for 12 consecutive days. At the end of the observation period post-challenge, the surviving birds were sacrificed to determine the bacterial load in liver and spleen as previously described [28].

### Systemic IgG and mucosal IgA specific antibody responses

The systemic M2e and SG specific IgG responses were analysed in vaccinated sera by an indirect ELISA as described previously [28]. For analysis of mucosal M2e and SG specific IgA responses, three birds in each group were sacrificed at 28^th^ day post-immunization and intestinal and lung wash samples were collected for determination of IgA titers by an indirect ELISA. Purified M2e (250 ng/well) and SG ompA (250 ng/well) proteins were used as coating antigens for ELISA and for the stimulation of PBMCs in vitro.

### Antigen-specific cellular immune responses

The cell mediated immunity elicited by vaccination was evaluated by a lymphocyte proliferation test. Two weeks post-vaccination, in vitro proliferative capacity of vaccinated PBMCs (1 × 10^6^, *n* = 5) in response to the recall M2e or ompA antigen was determined by a MTT [3-(4,5-dimethylthiazol-2-yl)-2,5-diphenyltetrazolium bromide] based assay [21].

For analysis of IFN-γ responses, PBMCs (1 × 10^6^, *n* = 5) isolated from vaccinated and non-vaccinated birds restimulated with either M2e (10 µg/mL) or ompA (10 µg/mL) recall antigen were harvested after 24 h incubation at 37 °C in 5% CO_2_ and then total RNA isolated from stimulated cells was analysed for IFN-γ mRNA transcription by qRT-PCR assay [21].

### Statistical analysis

All the obtained data was analysed using GraphPad prism 6.00 program (San Diego, CA, USA). Statistical significance was determined by t-tests (two-tailed) for two groups or one-way ANOVA (with Tukey’s multiple comparisons tests) for more than two groups. A non-parametric Chi square test was used to analyze significant differences in mortality. *p* values of < 0.05 were considered statistically significant.

## Results

### Attenuated SG efficiently expressed M2eCD40L fusion protein

**Figure 1:**
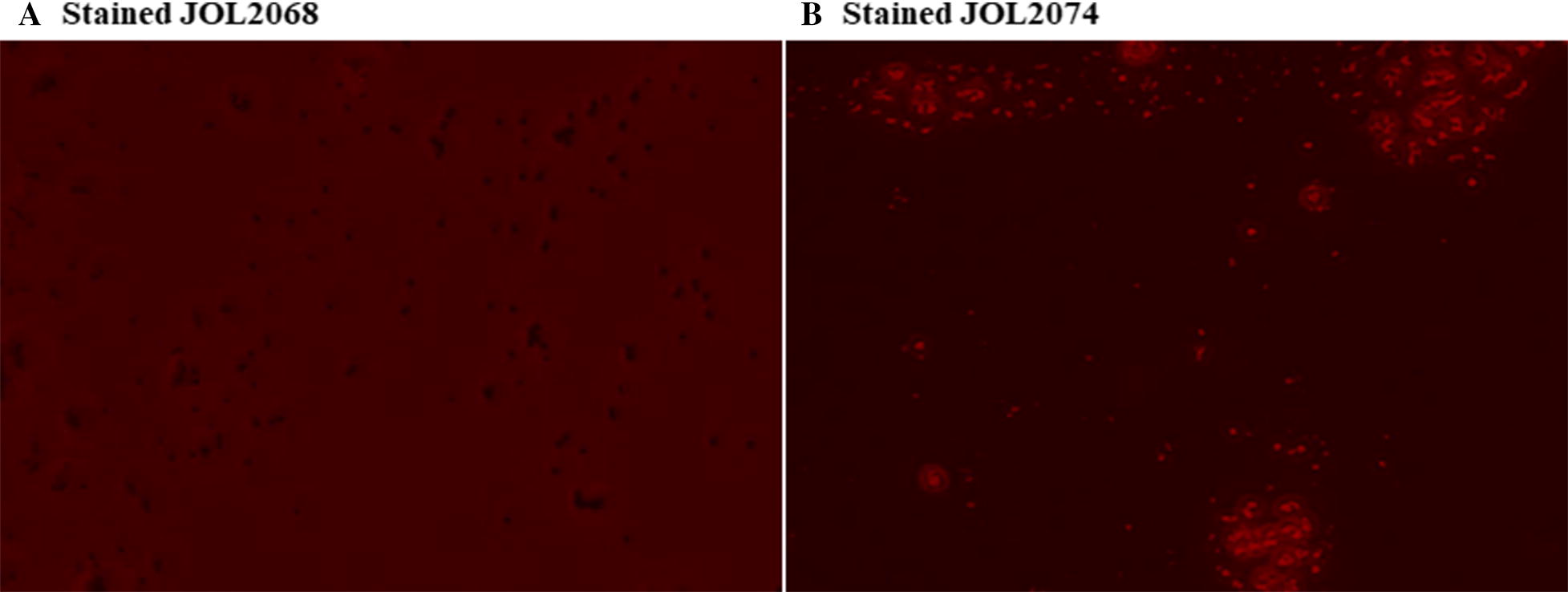
**M2eCD40L expression in SG.** The M2eCD40L gene expression in SG was confirmed by an indirect immunofluorescence using primary M2-specific polyclonal antibody, followed by labelling of bacterial cells with PE-conjugated species-specific secondary antibody. **A** JOL2068 bacteria lacking M2e gene. **B** JOL2074 bacteria expressing M2e protein as indicated by an immunofluorescence.

To construct the SG-M2eCD40L based influenza vaccine, the synthesized M2eCD40L gene sequence was cloned in frame either into pJHL65 prokaryotic expression vector or pcDNA3.1+ mammalian expression vector. The presence of M2eCD40L gene in pJHL65 and pcDNA3.1 recombinant plasmids was confirmed by digestion of the recombinant clones with restriction-specific enzymes to release a fragment of 123 bp size. The recombinant plasmids pJHL-M2eCD40L and pcDNA-M2eCD40L were transformed into JOL967 and JOL2068 strains, respectively, for expression and delivery of M2eCD40L as described previously [22]. The display of M2eCD40L protein expressed by pJHL65 vector on the surface of SG was confirmed by an indirect immunofluorescence (IF) method and flow cytometry. Our IF results indicated that recombinant SG bacteria (JOL2074) efficiently displayed surface M2e expression as evidenced by the reactivity of the M2-specific polyclonal antibody with the expressed M2eCD40L protein, which was lacking in the negative bacterial control, JOL2068 strain (Figures 1A and B). The M2eCD40L expression in SG was further confirmed by flow-cytometry. Our flow cytometric results showed that JOL2074 recombinant bacteria efficiently expressed M2eCD40L fusion protein as evidenced by the dramatic increase in fluorescence post-staining with M2-specific antibody compared to the JOL2068 strain that showed very little non-specific fluorescence (Additional file 1). This finding was in accord to the previously published report [25]. We also cloned and expressed the M2eCD40L protein in *E. coli* BL21 host strain for in vitro studies. As shown in Additional file 2, the Coomassive brilliant blue stained gel showed polyHis6-tagged M2eCD40L fusion protein to be ~ 24 kDa, the expected size of our protein of interest in pET32a vector, thus confirmed the M2eCD40L expression.

To investigate whether the presence of M2eCD40L has any effect on the growth of SG, the growth kinetics of JOL2068, JOL2074 and JOL2076 were compared by allowing each bacterial strain to grow in LB broth at 37 °C. Our results indicated that the delivery of M2eCD40L either in protein or in DNA format did not result in an impaired viability of the bacterial carrier and all the strains showed similar growth kinetics (Additional file 3).

### Attenuated SG efficiently delivered pcDNA-M2eCD40L DNA plasmid into mammalian cells

**Figure 2:**
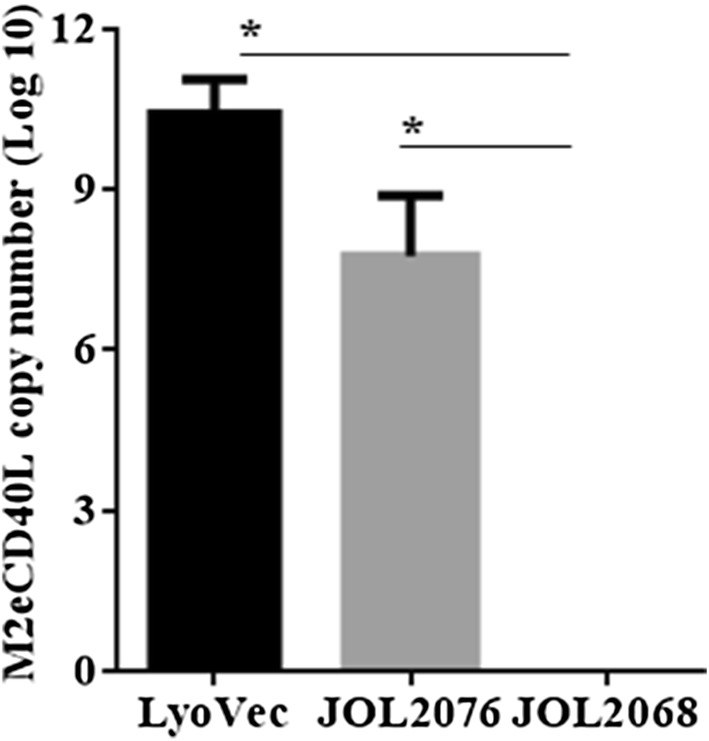
**SG mediated delivery of M2eCD40L DNA plasmid into MDCK cells.** The potential of SG to deliver pcDNA-M2eCD40L DNA plasmid into mammalian cells was evaluated in cultured MDCK cells infected with either JOL2068 or JOL2076 (20 bacteria/cell). LyoVec mediated transfer of M2eCD40L DNA plasmid into cells was kept as a positive control. The M2eCD40L gene expression was analysed at mRNA level by calculating M2eCD40L RNA copy number. Data is represented in log_10_ M2eCD40L copy number. Statistical significance was determined by Student’s *t*-test (two-tailed). *p < 0.05.

To determine whether *Salmonella* can efficiently deliver the pcDNA plasmid encoding M2eCD40L into mammalian cells, MDCK cells were infected with either JOL2076 carrying a pcDNA-M2eCD40L plasmid or JOL2068 carrying an empty vector, and harvested at 30 h post-infection for analysis of M2eCD40L expression at mRNA level by qRT-PCR assay (Figure 2). We compared the expression levels of M2eCD40L delivered by SG to that of the LyoVec mediated transfer of pcDNA-M2eCD40L into MDCK cells. Our results showed that JOL2068, which carried an empty vector, did not show any M2eCD40L mRNA expression. The M2eCD40L mRNA expression levels were detected in cells treated with either JOL2076 or LyoVec-pcDNAM2eCD40L complex (Figure 2). Although SG mediated efficient delivery of pcDNA plasmid into mammalian cells, however, the delivery of DNA was significantly (*p* < 0.001) lower compared to the LyoVec. The expression levels in LyoVec were 1000 folds higher than SG mediated gene transfer. These results, thus, indicate that *Salmonella* can carry out delivery and expression of constructs containing DNAs encoding for viral antigens.

### Coadministration of JOL2074 and JOL2076 strains induce efficient M2e and SG specific systemic and mucosal antibody responses

**Figure 3:**
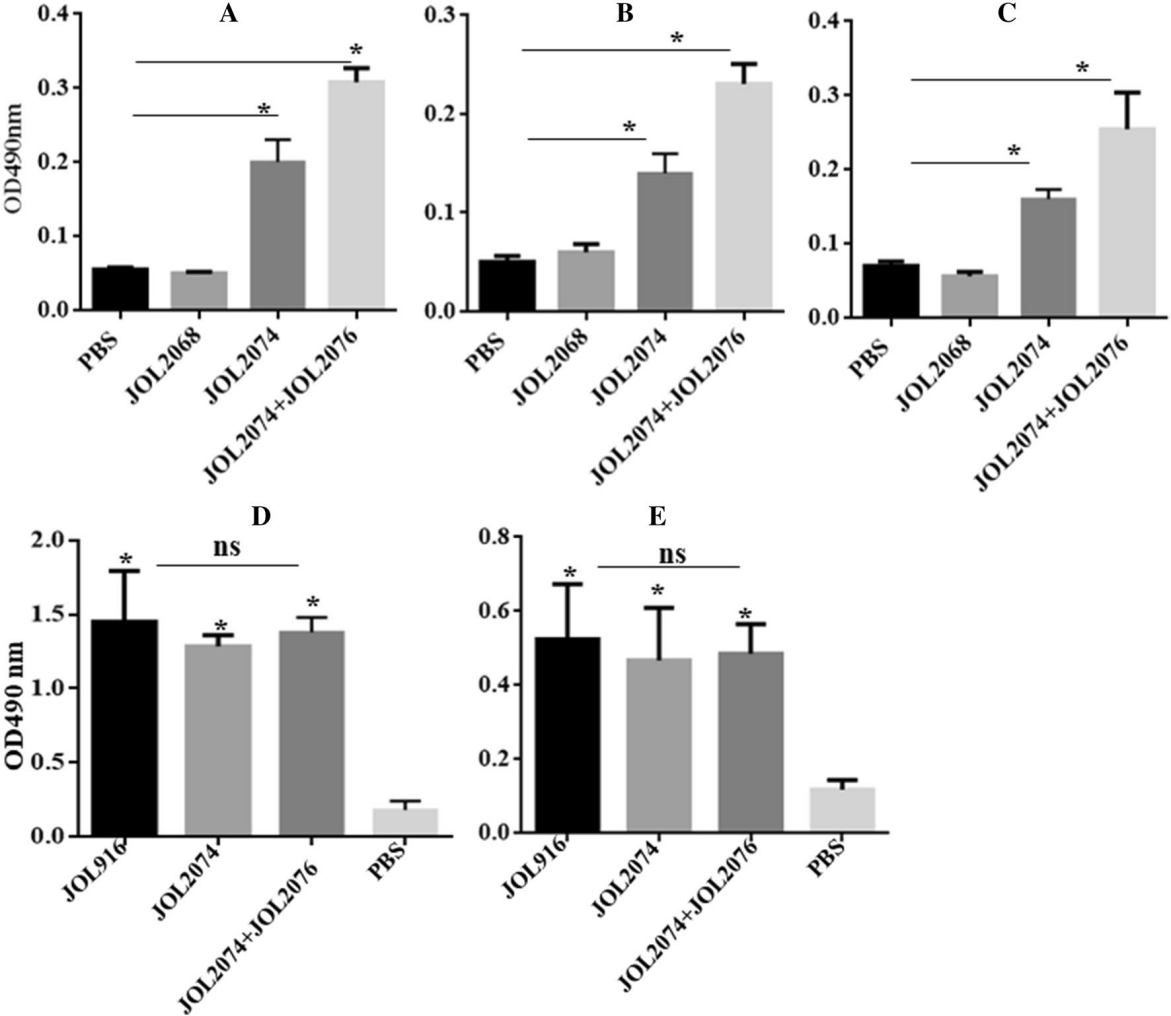
**SG-based M2eCD40L vaccine elicits M2e and SG specific systemic IgG and mucosal IgA responses.** The serum IgG (1:100 dilution) and mucosal IgA (1:30 dilution) responses in intestinal and lung wash samples were analysed by an indirect ELISA. The antibody responses were measured at 28^th^ day post-vaccination. **A** M2e-specific serum IgG responses. **B** M2e-specific intestinal sIgA responses. **C** M2e-specific lung sIgA responses. **D** ompA-specific serum IgG responses. **E** ompA-specific intestinal sIgA responses. Each data points represent mean ± SD of five chickens per group for IgG analysis and three chickens for IgA analysis. Statistical significance was determined by one-way ANOVA (with Tukey’s multiple comparisons tests). *p < 0.05. ns: non-significant.

To investigate the effect of immunization on the systemic and mucosal antibody responses, we vaccinated chickens with JOL2074, JOL2074 plus JOL2076 or JOL2068, and measured IgG in serum and secretory (s) IgA in intestines and in lungs at 28^th^ day post-immunization by an indirect ELISA. Chickens vaccinated with either JOL2074 or JOL2074 plus JOL2076 showed significantly (*p* < 0.05) higher M2e-specific IgG and IgA levels compared to the chickens that received JOL2068 strain (Figures 3A–C). Among groups that received M2eCD40L vaccine, chickens vaccinated with a mixture of JOL2074 and JOL2076 strains elicited significantly (*p* < 0.05) higher both IgG and IgA responses compared to the chickens vaccinated with JOL2074 alone, suggesting that coadministration of M2eCD40L in protein and in DNA format induces efficient induction of systemic and mucosal humoral immune responses.

We also measured SG-specific IgG and sIgA responses in serum and in intestines, respectively. We found significantly (*p* < 0.05) higher IgG and IgA responses in chickens vaccinated with either JOL2074 or JOL2074 + JOL2076 compared to the PBS control group (Figures 3D and E). The SG-specific IgG and IgA responses induced by SG-M2eCD40L based vaccines were almost comparable to that of the chickens vaccinated with JOL916 mutant strain, suggesting that SG can be exploited to deliver foreign antigens without affecting the SG-specific immunity.

### Co-immunization with JOL2074 and JOL2076 elicited efficient M2e and SG-specific cellular immunity

**Figure 4:**
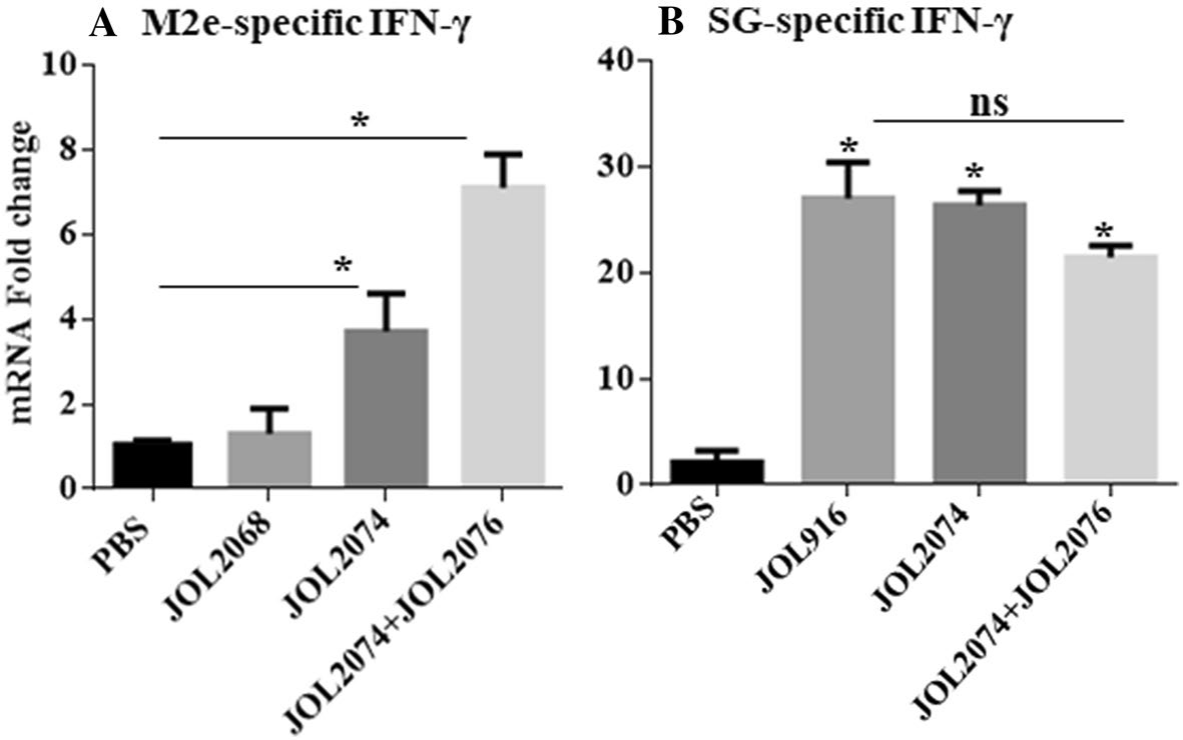
**In vitro proliferations of lymphocytes from vaccinated chickens in response to recall M2e or ompA antigen.** Chickens (*N* = 10) were vaccinated with JOL916, JOL2068, JOL2074 or JOL2074 + JOL2076, and PBMCs from vaccinated chickens after 14 days post-immunization were restimulated with either M2e or ompA (10 µg/mL) for 72 h and lymphocyte proliferation was determined by MTT-based assay. Results are expressed as stimulation indices, defined as proliferations in response to recall antigen relative to the mock stimulated cells. **A** M2e-specific lymphocyte proliferation assay. **B** ompA-specific lymphocyte proliferation assay. Each data points represent mean ± SD of five chickens per group. Statistical significance was determined by one-way ANOVA (with Tukey’s multiple comparisons tests). *p < 0.05. ns: non-significant.

**Figure 5:**
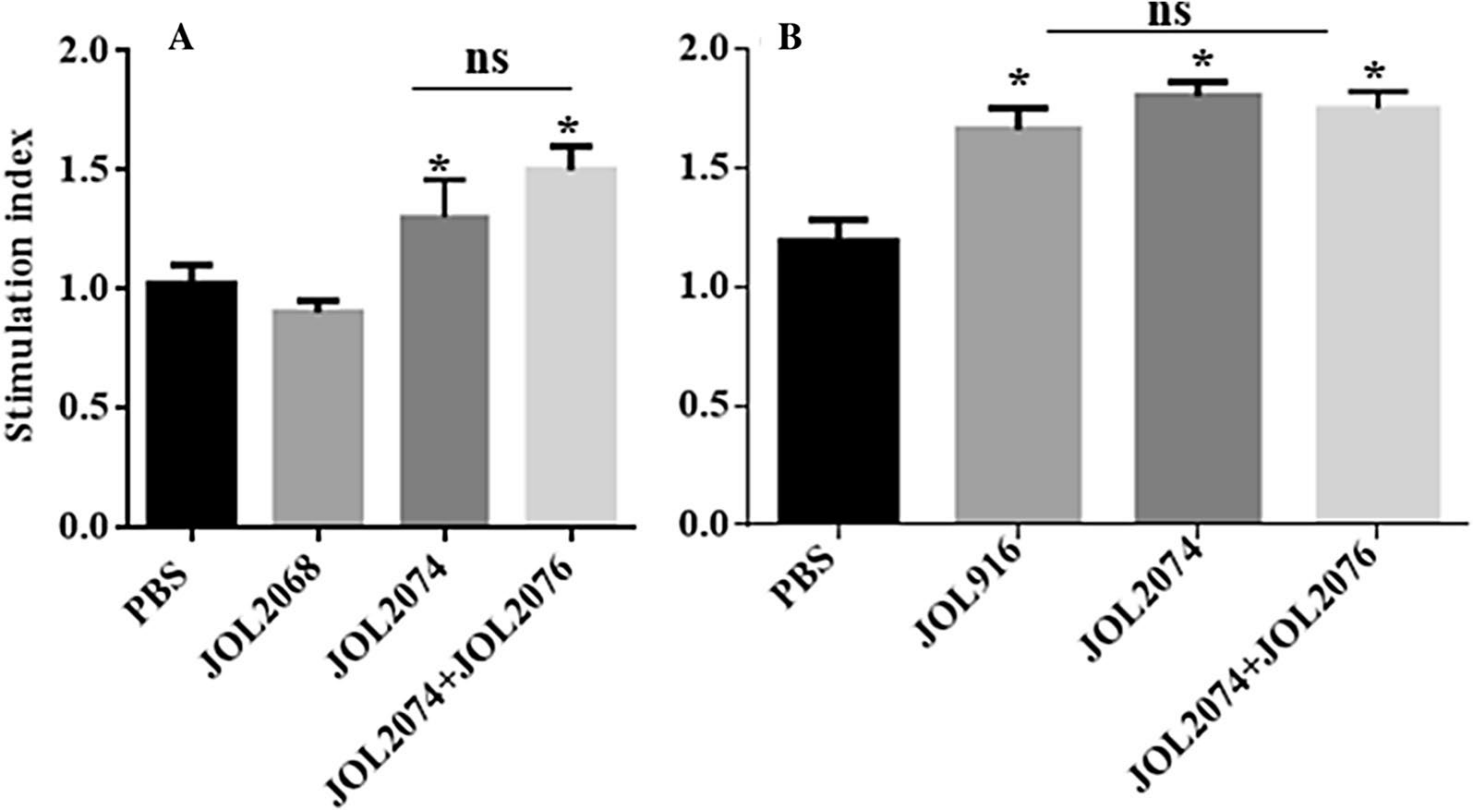
**qRT-PCR analysis of IFN-γ responses in vaccinated PBMCs after stimulation with either M2e or ompA recall antigen.** Chickens (*N* = 10) were vaccinated with JOL916, JOL2068, JOL2074 or JOL2074 + JOL2076, and PBMCs from vaccinated chickens after 14 days post-immunization were stimulated with either M2e or ompA recall antigen for 24 h and analysed for induction of IFN-γ mRNA transcription by qRT-PCR assay. Results are expressed as fold change in mRNA transcription after antigenic stimulation of immunized PBMCs compared to the naïve PBMCs treated with M2e or ompA antigen. Gene expressions were normalized to GAPDH and mRNA levels at 0 h was used as calibrator. **A** M2e-specific IFN-γ responses. **B** ompA-specific IFN-γ responses. Data presented are mean ± SD of five chickens per group. Statistical significance was determined by one-way ANOVA (with Tukey’s multiple comparisons tests). *p < 0.05, ns: non-significant.

To assess the effect of vaccination on the M2e-specific cellular immune responses, we isolated PBMCs from vaccinated chickens and restimulated with M2e antigen in vitro for analysis of IFN-γ and lymphocyte proliferative responses by qRT-PCR and MTT assay, respectively (Figures 4 and 5). Our results indicated that the chickens vaccinated with JOL2074 or JOL2074 plus JOL2076 showed significantly (*p* < 0.05) higher IFN-γ mRNA levels compared to the JOL2068 vaccinated group (Figure 4A). Among chickens that received M2eCD40L-based vaccine, IFN-γ were significantly (*p* < 0.05) higher (twofolds) in JOL2074 + JOL2076 vaccinated group compared to the JOL2074 alone group (Figure 4A). We also measured T cell responses by a MTT based assay. While T cell proliferative responses were significantly (*p* < 0.05) higher in both JOL2074 and JOL2074 plus JOL2076 vaccinated groups compared to the JOL2068 control group, however, the lymphoproliferative responses were slightly higher in chickens that received a co-mix of JOL2074 and JOL2076 strains (Figure 5A). These results indicate that co-immunization with SG strains delivering M2eCD40L in protein and in DNA format enhanced M2e-specific humoral and cellular immune responses.

Next we measured SG-specific cell mediated immune (CMI) responses following stimulation of vaccinated and naïve PBMCs with ompA recall antigen (Figures 4 and 5). Our results demonstrated that vaccination with JOL916, JOL2074 or JOL2074 plus JOL2076 induced comparable but significantly (*p* < 0.05) higher IFN-γ mRNA levels (tenfolds higher) (Figure 4B) and proliferative T cell responses (Figure 5B) compared to the chickens that received PBS only. Thus, our results clearly suggest that SG delivering foreign antigens can efficiently induce CMI responses against both the carrier and the delivered heterologous antigens.

### Immunization with attenuated SG delivering M2eCD40L provided efficient protection against H9N2 and wild-type SG infection

**Figure 6:**
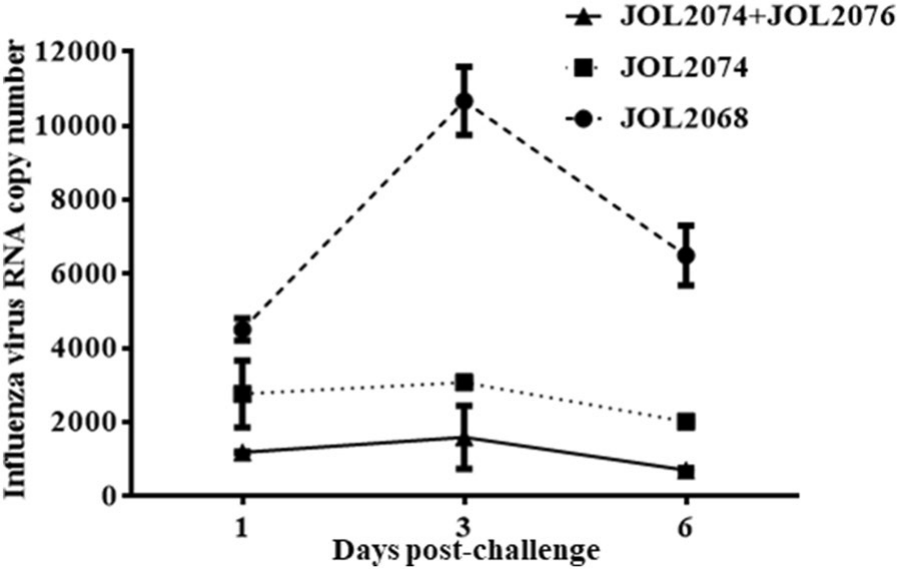
**Protective efficacies of the SG-based M2eCD40L vaccine against LPAI H9N2 challenge.** Chickens (*N* = 10) were vaccinated with JOL2068, JOL2074 or JOL2074 + JOL2076, and 28 days later all the vaccinated chickens were challenged with 10^5^ TCID_50_ H9N2 virus. The protective efficacy was determined by estimation of H9N2 viral RNA copy numbers in the cloacal swab samples of the immunized chickens (*n* = 4) after challenge with the virulent H9N2 virus. The experiment was repeated twice and the results are shown of one independent experiment.

**Figure 7:**
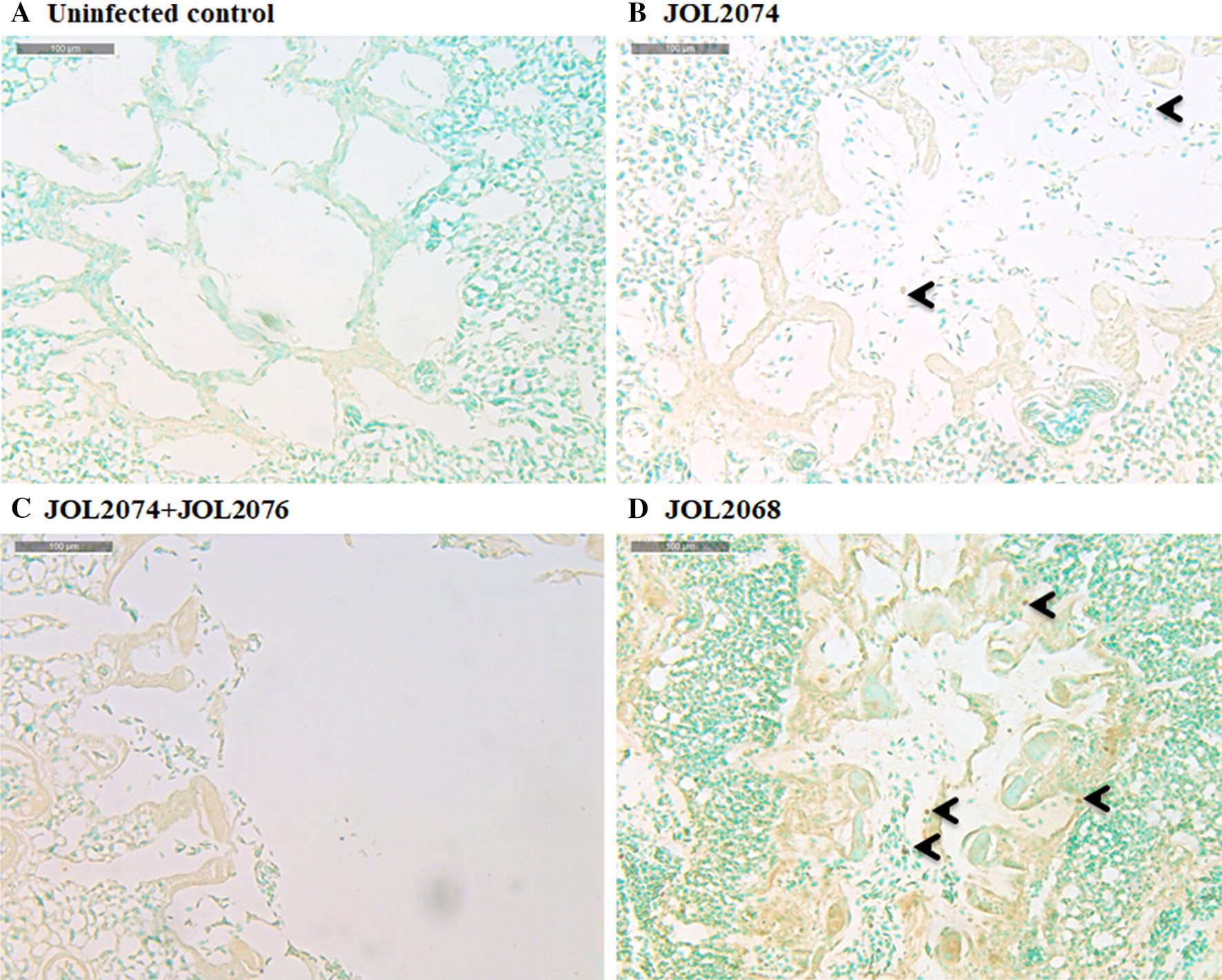
**Photomicrographs of Methyl Green-stained lung sections of chickens on day 3 post-H9N2 challenge.** Chickens (*N* = 10) were vaccinated with JOL2068, JOL2074 or JOL2074 + JOL2076, and 28 days later all the vaccinated chickens were challenged with 10^5^ TCID_50_ H9N2 virus. At 3^rd^ day post-challenge, chickens (*n* = 3) were sacrificed and lung tissues were collected for immunohistochemistry analysis. The experiment was repeated twice and the results are shown of one independent experiment. Brown dots indicated by arrows represent H9N2 virus particles.

To determine the protective efficacy of a SG-based M2e vaccine against influenza, we intranasally challenged all the vaccinated and the control chickens with a virulent dose of 10^5^ TCID_50_ H9N2 virus. Subsequently, cloacal swab samples were collected post-challenge for the determination of viral RNA copy number by qRT-PCR assay (Additional file 4). The presence of viral RNA was found in all the groups from day 1 to 6, albeit, chickens immunized with JOL2074 or JOL2074 + JOL2076 showed significantly (*p* < 0.05) lower viral load compared to the control chickens (Figure 6). Among immunized groups, chickens which received a co-mix of JOL2074 + JOL2076 strains showed significantly (*p* < 0.05) lower viral load from day 1 to day 6 compared to the JOL2074 vaccinated group (Figure 6). This finding was recapitulating with the immunohistochemistry results performed on lung tissues collected from vaccinated and control birds on day 3 post-challenge (Figure 7). Immunohistochemistry results revealved that the birds vaccinated with JOL2074 + JOL2076 had significantly (*p* < 0.05) lower viral load compared to the JOL2074 and JOL2067 vaccinated groups, suggesting that SG delivering M2eCD40L in both protein and DNA forms efficiently contained the H9N2 infection and thus considerably reduced the viral shedding in birds.

**Figure 8:**
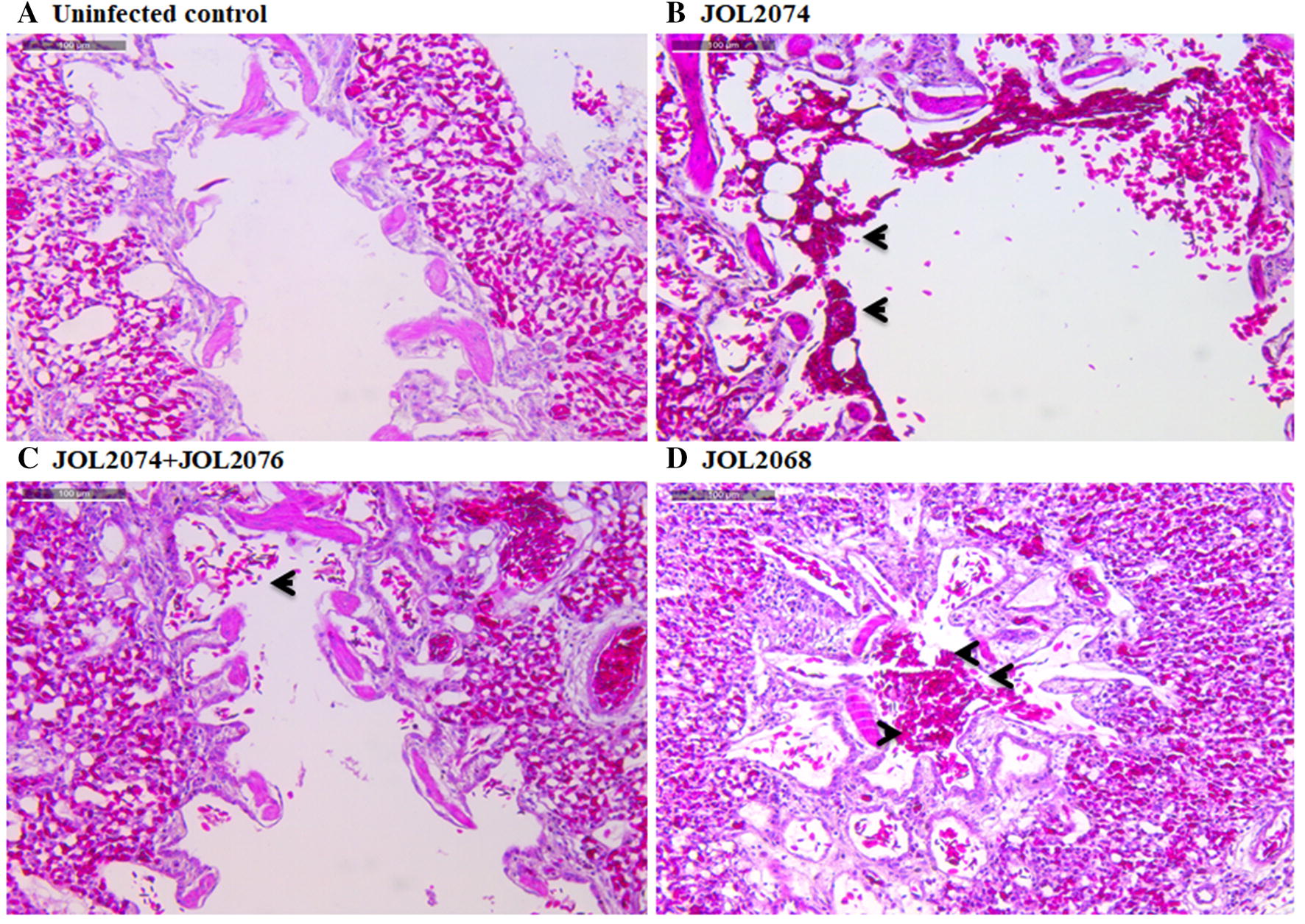
**Photomicrographs of hematoxylin-and eosin-stained lung sections of chickens on 7**^th^ **day post-H9N2 challenge.** Chickens (*N* = 10) were vaccinated with JOL2068, JOL2074 or JOL2074 + JOL2076, and 28 days later all the vaccinated chickens were challenged with 10^5^ TCID_50_ H9N2 virus. At 7^th^ day post-challenge, chickens (*n* = 4) were sacrificed and lung tissues were collected for histological analysis. Chickens that received JOL2068 showed hyperemia with infiltration of mononuclear inflammatory cells in bronchi and lung parenchyma, while as vaccinated birds showed minimum inflammatory lesions. Arrows indicate inflammatory lesions. The experiment was repeated twice and the results are shown of one independent experiment.

To further investigate the effect of M2eCD40L vaccination on virus-specific immune protection, histopathological studies were performed on lung tissues collected from birds on day 3 and 7 post-H9N2 challenge (Figure 8). As expected, no lesion was found in the lung tissues of uninfected chicken. Chickens treated with control JOL2068 strain had pulmonary lesions consisting of inflammation and hemorrhagic exudates in the lungs. The pulmonary lesions were more profound on day 7 post-challenge (Figure 8) compared to the day 3 post-challenge (Additional file 5). Chickens vaccinated with JOL2074 + JOL2076 lacked lesions in lungs compared to the JOL2074 vaccination that exhibited minimal inflammation. Further, lungs isolated from JOL2068, which served as control, showed reddish discoloration, indicating a hemorrhagic effect characteristic to the influenza infection (Additional file 6). The general appearance of the birds post-H9N2 challenge was observed for seven consecutive days. Compared to the vaccinated birds, control birds showed disease symptoms including ruffled feathers, depression, anorexia and even some birds exhibited diarrhea. These signs started from day 3 onwards post-challenge. In contrast, no clinical signs were observed in both of the M2eCD40L vaccinated-challenged groups.

We also investigated the protective efficacy of SG-based M2e vaccine against FT by orally challenging vaccinated and control chickens with a lethal dose (1.6 × 10^6^ CFU) of wild-type SG bacteria. The mortality of birds, index of protection efficacy in this study, was observed for 12 days post-challenge and the results are shown in Table 2. The chickens immunized with JOL916, JOL2074 or JOL2074 + JOL2076 exhibited lower mortality rate (12.5%, 1 of 8 birds died) compared to the control chickens, which showed 75% mortality (6 of 8 birds died). The efficacy of SG-M2eCD40L based vaccine was further evaluated by post-mortem analysis of the survived birds at 12 days post-challenge. Our results showed that the birds vaccinated with either JOL916 or SG-M2eCD40L vaccines had significantly (*p* < 0.05) lower bacterial load in spleen and liver compared to the control group (Table 2).

**Table 2:** Mortality and bacterial load in the chickens post-challenge

Groups^a^	Challenge^b^			Liver^e^	Spleen^e^
	Mortality^c^	Liver^d^	Spleen^d^		
PBS	6/8*	2/2*	2/2*	3.44 ± 2.42*	2.8 ± 1.84*
JOL916	1/8	4/7	0/7	2.88 ± 2.13	0
JOL2074	1/8	5/7	1/7	2.92 ± 2.20	0.75 ± 1.17
JOL2074 + JOL2076	1/8	4/7	0/7	2.75 ± 2.09	0

^a^Four weeks old chickens (*n* = 8) were orally vaccinated with PBS, JOL916, JOL2074 or JOL2074 + JOL2076.

^b^Challenge was performed with a wild-type SG strain using 1.6 × 10^6^ CFU/bird after 28 days post-vaccination.

^c^Number of dead birds upon challenge.

^d^Number of birds positive for SG.

^e^Bacterial load in liver (CFU/gram) and spleen (CFU/spleen). *p < 0.05.

## Discussion

The present study was aimed to investigate the protective efficacy of a SG-based M2e vaccine against FT and H9N2 infection in chickens. A major obstacle in influenza vaccine development is the extent of genetic diversity among influenza subtypes and therefore immunity provided by one vaccine strain provides little or no protection against the heterologous strain. M2e is a promising vaccine candidate to induce broad spectrum of protection against many subtypes as it is highly conserved across influenza A viruses [15]. Several considerable studies have demonstrated that M2e conjugated to various carrier molecules enhanced immunogenicity as well as cross-protective immunity of M2e-based vaccines in various animal models (reviewed in [15]). Therefore, in this study, we physically linked M2e to CD40L and delivered this fusion gene via SG. Furthermore, M2e has been included in different DNA vaccine formats to induce broadly protective anti-influenza antibodies; however, such approaches yielded low M2e-specific antibody responses [29–31]. To elicit effective M2e-specific immunity, we delivered M2eCD40L in both protein and DNA forms via SG in this study. We show that chickens immunized with a co-mix of SG mutant strains delivering M2eCD40L in both protein and DNA formats elicited efficient M2e-specific immune responses compared to the chickens that received M2eCD40L in protein format only.

Live attenuated *Salmonella* vaccine vectors expressing recombinant heterologous antigens have previously been shown to elicit humoral and CMI responses against both *Salmonella* and foreign antigens, and attenuated *Salmonella* mutant strains have long been approved for use in human and veterinary medicine [32]. *S.* Typhimurium system has previously been utilized to deliver influenza A viral antigens and these studies have demonstrated efficient induction of influenza-specific immunity and subsequent protection against lethal challenges [20–22, 33]. Induction of M2e-specific IgG antibody responses in peripheral blood circulation is essential for protection against influenza A viruses mediated by M2e-based vaccines [34]. A previous report by Song et al. [35] shows that prophylactic and therapeutic administration of M2e-specific Z3G1 monoclonal antibody resulted in significant protection in mice and alleviated clinical symptoms and lung pathology in monkeys following H1N1 infection. The present study demonstrated that codelivery of SG mutant strains expressing and delivering M2eCD40L in protein and DNA formats induced elicited levels of M2e IgG responses and subsequent protection against the H9N2 challenge. The IgG responses were more robust in JOL2074 plus JOL2076 group compared to the JOL2074 alone group. Consequently, the protection rate, in the context of viral load and lung pathology, was lower in JOL2074 plus JOL2076 vaccinated group. This clearly indicates that coadministration of protein and DNA formats of M2e acted synergistically to induce efficient immune responses against H9N2 infection. The induction of anti-M2e IgA responses also plays a critical role in protection mediated by M2e-based vaccines. A previous report shows that intranasally administered M2e based vaccine induced better protection than subcutaneously immunized mice against PR8 virus challenge [36]. The present study demonstrated that codelivery of JOL2074 plus JOL2076 strains elicited higher intestinal and lung IgA responses compared to the JOL2074 alone immunized group. This finding might explain the higher immune protection rate observed in JOL2074 plus JOL2076 immunized chickens. Besides humoral immunity, there are several studies that show T cell responses also play an important role in influenza protection conferred by M2e-based vaccines. Pejoski et al. [37] reported that immunization with Freund’s adjuvanted M2e2-16 peptide containing only B cell epitopes failed to induce antibody responses; however, M2e-specific antibodies are readily generated by including a chemically conjugated T helper epitopes derived from HA in the M2e conjugate, indicating that T cells plays an important role in the M2e mediated immunity. Further, Eliasson et al. [38] shows that M2e-specific T cell responses are broadly protective against influenza infections and poor protection was observed in vaccinated mice that failed to develop CD4+ T cell responses following M2e based vaccination. The present study demonstrated that T cell proliferative responses were significantly higher in JOL2074 plus JOL2076 immunized group compared to the JOL2074 alone vaccinated chickens. We also observed significantly higher IFN-γ responses, a surrogate measure of T cell responses, in chickens vaccinated with a co-mix of JOL2074 and JOL2076 strains. Consequently, JOL2074 plus JOL2076 immunized chickens had shown lower viral load and lung pathology compared to the JOL2074 vaccinated group, indicating that T cells might play an important role in protection mediated by M2e-based vaccines. The M2e-specific T cells drive B cells to accelerate and generate more potent M2e and hemagglutinin-specific IgG production [38]. This reason might further explain why higher production of M2e-specfic IgG and IgA responses were observed in JOL2074 plus JOL2076 vaccinated chickens compared to the JOL2074 alone vaccinated group.

The present study further demonstrated that SG-based M2e vaccine can elicit efficient protection against FT as well. The induction of humoral and cellular immunity following vaccination strongly correlates with the recovery from FT and protection from subsequent challenge infection in chickens [19, 28]. The humoral responses, particularly the systemic IgG, mediate protection during the early stages of infection when SG bacterium circulates extracellularly before it penetrates the target cells [39]. The present study demonstrated that systemic IgG responses were significantly higher in chickens vaccinated with either SG or SG-M2e based vaccines compared to the control chickens. Consequently, the mortality rate was lower in vaccinated chickens compared to the chickens that received PBS only. The induction of immune responses at mucosal surfaces serves as the first line defense against pathogens that enter into systemic circulation via mucosal surfaces. Importantly, the presence of antigen-specific secretory (s) IgA antibodies in the intestinal mucus acts as an immunological barrier, thereby preventing the adherence of *Salmonella* bacteria to the intestinal lining and subsequent penetration into deeper tissues [40]. In the present study, we observed that the sIgA levels were significantly higher but comparable in all the immunized chicken groups compared to the control group. Thus, protection against the wild-type SG was similar in chickens vaccinated with either SG or SG-M2e based vaccines. SG is an intracellular bacterium, and survives and replicates in macrophages [41]. Therefore, the induction of cell mediated immunity is important for bacterial clearance from systemic organs and subsequent recovery from the clinical disease. Earlier reports have shown strong correlation between induction of cell mediated immunity and survival of birds following lethal SG challenge [19, 41]. In the present study, the SG-based M2e vaccine not only generated efficient humoral responses but also stimulated high level of antigen-specific cellular responses. The present study demonstrated that immunization with either SG or SG-M2e based vaccines elicited significantly higher but comparable T cell proliferative responses compared to the PBS control group. We also observed IFN-γ, a Th1-type cytokine, responses higher in vaccinated groups than control chickens. IFN-γ is critical in the development of CMI responses, especially cytotoxic CD8+ responses, which are more potent and effective in clearance of intracellular infections [42, 43]. This might explain why immunized chickens cleared bacterial infection at a faster rate than PBS control group. These results point out that SG based M2e vaccine has potential to elicit SG and M2e-specific humoral and CMI responses, and can thus offer dual protection against FT and LPAI infection, as observed in this study.

In conclusion, we show that SG-based M2e vaccine can elicit antigen-specific humoral and CMI responses and can offer dual protection against both FT and H9N2 infection in chickens. Other studies have shown that efficacious LPAI and HPAI vaccines protect against clinical signs and mortality, reduce virus shedding, and increase resistance to infection. Our results clearly show that vaccination with SG-based M2eCD40L vaccine elicited efficient protection against H9N2 infection as evidenced by insignificant lung inflammation and reduced viral load in both cloacal samples and lungs. Further studies are warranted to develop this SG-M2eCD40L based vaccine as a broadly protective vaccine against avian influenza virus subtypes.

## Additional files

**Additional file 1.** **Analysis of M2eCD40L protein displayed on the surface of SG.** M2e gene was physically linked to chicken CD40L peptide and cloned into constitutive pJHL65 expression vector and the recombinant plasmid was subsequently electroporated into attenuated SG mutant strain, JOL2074. The bacterial M2eCD40L surface expression was analysed by flow cytometry using primary M2e-specific polyclonal antibody and then Alexa Fluor 488-conjugated species-specific secondary antibody body. (A) Gating of SG bacteria excluding debris and dead cells. (B) FACS histogram of unstained bacterial control, JOL2074 (B) FACS histogram of stained JOL2068 bacteria lacking M2e gene. (C) FACS histogram of stained JOL2074 bacteria expressing M2e protein showing dramatic increase in fluorescence.

**Additional file 2.** **SDS-PAGE analysis of M2eCD40L in** ***E. coli*** **BL21 host cells**. Lane 1, expression of M2eCD40L protein; lane 2, uninduced bacterial culture; lane M, protein marker (catalog#, P8500, GenDEPOT, USA).

**Additional file 3.** **In vitro analysis of growth kinetics of SG mutant strains.** To investigate the effect of M2eCD40L on the growth kinetics of SG mutant strain, bacterial strain carrying empty vector pJHL65, pJHL65-M2eCD40L or pcDNA-M2eCD40L was grown and OD600 nm was measured at different time points. The experiment was repeated twice and the results are shown of one independent experiment.

**Additional file 4.** **Challenge results of second experiment.** Three weeks old brown nick layer chickens (*N* = 10) were vaccinated with JOL2068, JOL2074 or JOL2074 + JOL2076, and 28 days later all the vaccinated chickens were challenged with 10^4^ TCID_50_ H9N2 virus. The protective efficacy was determined by estimation of H9N2 viral RNA copy numbers in the cloacal swab samples of the vaccinated chickens (*n* = 4) after challenge with the virulent H9N2 virus.

**Additional file 5.** **Photomicrographs of hematoxylin-and eosin-stained lung sections of chickens on 3**^rd^ **day post-H9N2 challenge**. Chickens (*N* = 10) were vaccinated with JOL2068, JOL2074 or JOL2074 + JOL2076, and 28 days later all the vaccinated chickens were challenged with 10^5^ TCID_50_ H9N2 virus. At 3^rd^ day post-challenge, chickens (*n* = 3) were sacrificed and lung tissues were collected for histopathological analysis. JOL2068 control chickens showed significantly higher inflammatory lesions compared to vaccinated JOL2074 and JOL2074 + JOL2076 chicken groups. Arrows indicate inflammation.

**Additional file 6.** **Morphological appearance of vaccinated chickens post-challenge with virulent H9N2 virus.** Chickens (*N* = 10) were vaccinated with JOL2068, JOL2074 or JOL2074 + JOL2076, and 28 days later chickens were challenged with 10^5^ TCID_50_ H9N2 virus. On 7^th^ day post-challenge, chickens (*n* = 4) were sacrificed and examined for gross lesions.
