# Supplementary figures and images for: Salmonella Gallinarum delivering M2eCD40L in protein and DNA formats acts as a bivalent vaccine against fowl typhoid and H9N2 infection in chickens
Source: Vet Res. 2018 Oct 1;49:99. doi: 10.1186/s13567-018-0593-z (PMC6389227; doi:10.1186/s13567-018-0593-z)

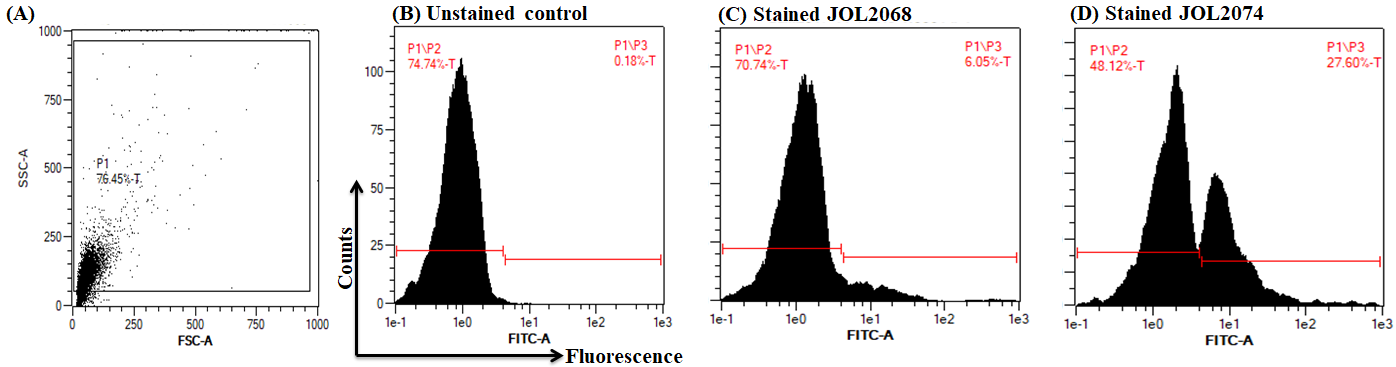

Supplement: Supplementary file 1 — Additional file 1. Analysis of M2eCD40L protein displayed on the surface of SG. M2e gene was physically linked to chicken CD40L peptide and cloned into constitutive pJHL65 expression vector and the recombinant plasmid was subsequently electroporated into attenuated SG mutant strain, JOL2074. The bacterial M2eCD40L surface expression was analysed by flow cytometry using primary M2e-specific polyclonal antibody and then Alexa Fluor 488-conjugated species-specific secondary antibody body. (A) Gating of SG bacteria excluding debris and dead cells. (B) FACS histogram of unstained bacterial control, JOL2074 (B) FACS histogram of stained JOL2068 bacteria lacking M2e gene. (C) FACS histogram of stained JOL2074 bacteria expressing M2e protein showing dramatic increase in fluorescence. [file 13567_2018_593_MOESM1_ESM.tif]

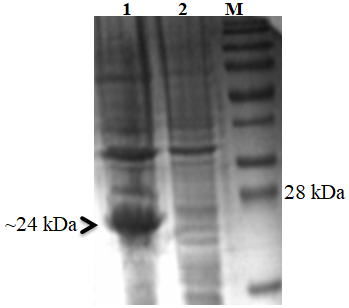

Supplement: Supplementary file 2 — Additional file 2. SDS-PAGE analysis of M2eCD40L in E. coli BL21 host cells. Lane 1, expression of M2eCD40L protein; lane 2, uninduced bacterial culture; lane M, protein marker (catalog#, P8500, GenDEPOT, USA). [file 13567_2018_593_MOESM2_ESM.tif]

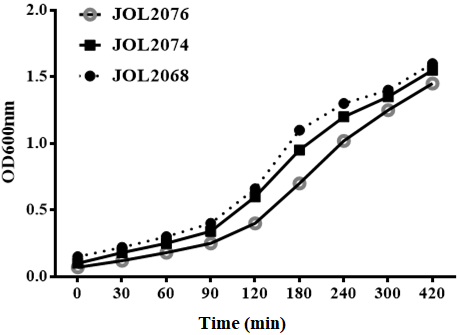

Supplement: Supplementary file 3 — Additional file 3. In vitro analysis of growth kinetics of SG mutant strains. To investigate the effect of M2eCD40L on the growth kinetics of SG mutant strain, bacterial strain carrying empty vector pJHL65, pJHL65-M2eCD40L or pcDNA-M2eCD40L was grown and OD600 nm was measured at different time points. The experiment was repeated twice and the results are shown of one independent experiment. [file 13567_2018_593_MOESM3_ESM.tif]

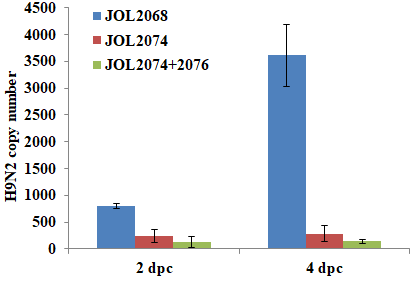

Supplement: Supplementary file 4 — Additional file 4. Challenge results of second experiment. Three weeks old brown nick layer chickens (N = 10) were vaccinated with JOL2068, JOL2074 or JOL2074 + JOL2076, and 28 days later all the vaccinated chickens were challenged with 104 TCID50 H9N2 virus. The protective efficacy was determined by estimation of H9N2 viral RNA copy numbers in the cloacal swab samples of the vaccinated chickens (n = 4) after challenge with the virulent H9N2 virus. [file 13567_2018_593_MOESM4_ESM.tif]

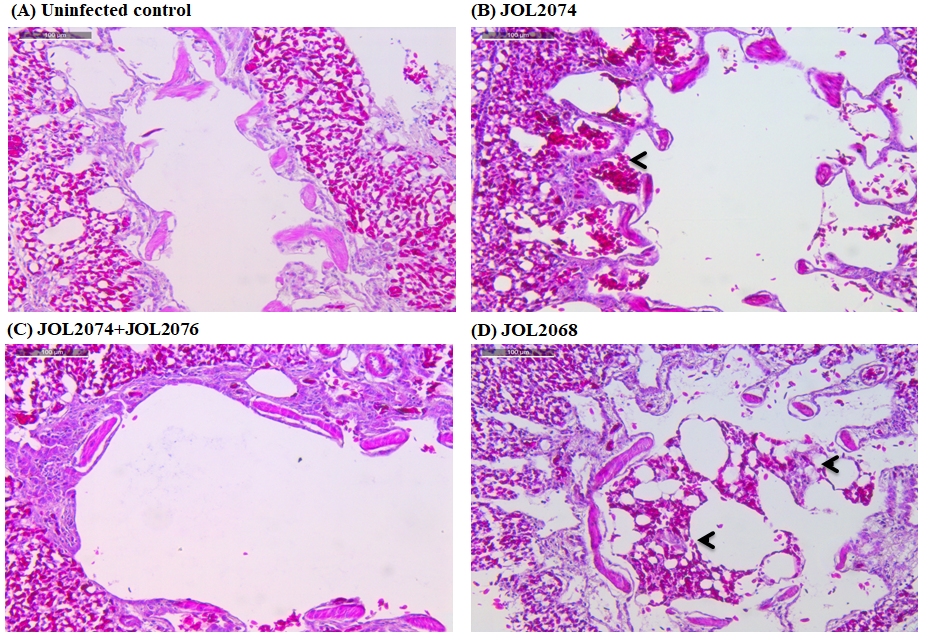

Supplement: Supplementary file 5 — Additional file 5. Photomicrographs of hematoxylin-and eosin-stained lung sections of chickens on 3rd day post-H9N2 challenge. Chickens (N = 10) were vaccinated with JOL2068, JOL2074 or JOL2074 + JOL2076, and 28 days later all the vaccinated chickens were challenged with 105 TCID50 H9N2 virus. At 3rd day post-challenge, chickens (n = 3) were sacrificed and lung tissues were collected for histopathological analysis. JOL2068 control chickens showed significantly higher inflammatory lesions compared to vaccinated JOL2074 and JOL2074 + JOL2076 chicken groups. Arrows indicate inflammation. [file 13567_2018_593_MOESM5_ESM.tif]

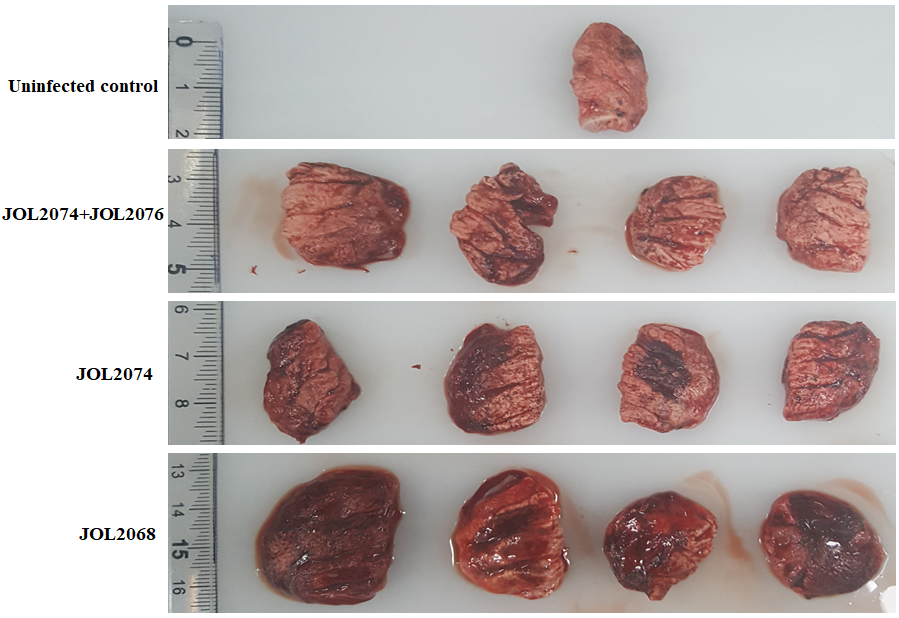

Supplement: Supplementary file 6 — Additional file 6. Morphological appearance of vaccinated chickens post-challenge with virulent H9N2 virus. Chickens (N = 10) were vaccinated with JOL2068, JOL2074 or JOL2074 + JOL2076, and 28 days later chickens were challenged with 105 TCID50 H9N2 virus. On 7th day post-challenge, chickens (n = 4) were sacrificed and examined for gross lesions. [file 13567_2018_593_MOESM6_ESM.tif]
